# Providing differentiated service delivery to the ageing population of people living with HIV

**DOI:** 10.1002/jia2.26002

**Published:** 2022-09-29

**Authors:** Catherine Godfrey, Snigdha Vallabhaneni, Minesh Pradyuman Shah, Anna Grimsrud

**Affiliations:** ^1^ Office of the Global AIDS Coordinator, Department of State Washington DC USA; ^2^ Division of Global HIV and TB, U.S Centers for Disease Control and Prevention Atlanta Georgia USA; ^3^ HIV Programmes and Advocacy, IAS – the International AIDS Society Cape Town South Africa

**Keywords:** ageing, differentiated service delivery, person‐centred care, co‐morbidities integration, PEPFAR, HIV/AIDS

## Abstract

**Introduction:**

Differentiated service delivery (DSD) models for HIV are a person‐centred approach to providing services across the HIV care cascade; DSD has an increasing policy and implementation support in high‐burden HIV countries. The life‐course approach to DSD for HIV treatment has focused on earlier life phases, childhood and adolescence, families, and supporting sexual and reproductive health during childbearing years. Older adults, defined as those over the age of 50, represent a growing proportion of HIV treatment cohorts with approximately 20% of those supported by PEPFAR in this age band and have specific health needs that differ from younger populations. Despite this, DSD models have not been designed or implemented to address the health needs of older adults.

**Discussion:**

Older adults living with HIV are more likely to have significant co‐morbid medical conditions. In addition to the commonly discussed co‐morbidities of hypertension and diabetes, they are at increased risk of cognitive impairment, frailty and mental health conditions. Age and HIV‐related cognitive impairment may necessitate the development of adapted educational materials. Identifying the optimal package of differentiated services to this population, including the frequency of clinical visits, types and location of services is important as is capacitating the healthcare cadres to adapt to these challenges. Technological advances, which have made remote monitoring of adherence and other aspects of disease management easier for younger populations, may not be as readily available or as familiar to older adults. To date, adaptations to service delivery have not been scaled and are limited to nascent programmes working to integrate treatment of common co‐morbidities.

**Conclusions:**

Older individuals living with HIV may benefit from a DSD approach that adapts care to the specific challenges of ageing with HIV. Models could be developed and validated using outcome measures, such as viral suppression and treatment continuity. DSD models for older adults should consider their specific health needs, such as high rates of co‐morbidities. This may require educational materials, health worker capacity building and outreach designed specifically to treat this age group.

## 1 INTRODUCTION

**Figure 1:**
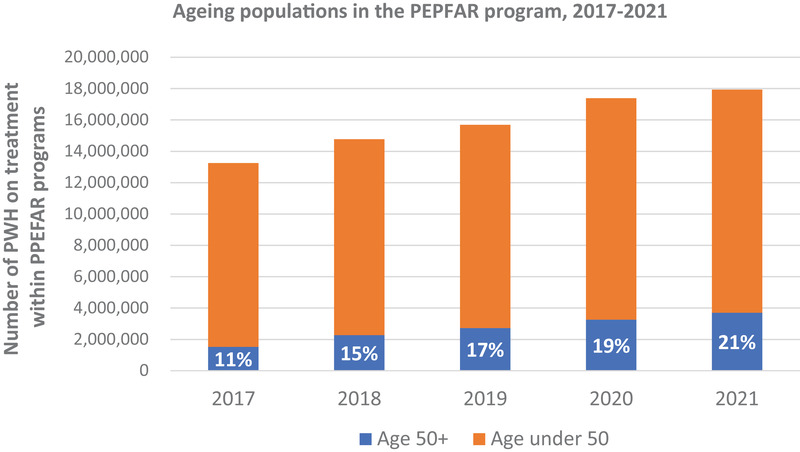
The number of people on HIV treatment in PEPFAR‐supported countries, 2017–2021, among older adults and those under 50 years of age. Between 2017 and 2021, the proportion of the HIV treatment cohort 50 years of age and above has increased from 11% to 21%. Abbreviation: PWH, people living with HIV.

The President's Emergency Program for AIDS Relief (PEPFAR) is a U.S. Government programme launched in 2003 to address the growing HIV pandemic in areas of the world most affected by new infections and deaths from HIV [1]. The programme has grown rapidly in both scale and breadth of HIV treatment services offered over the last two decades, largely by prioritizing the identification of people living with HIV, linking them to HIV care and treatment, and ensuring treatment continuity to achieve and maintain viral suppression. By the end of the fiscal year 2021, the PEPFAR programme supported HIV treatment for nearly 19 million people with HIV across 50 countries, many of which are in Africa [2]. In 2021, among people accessing HIV services in PEPFAR‐supported countries, 3.6 million or 21% were older adults—defined as 50 years or older. Of this cohort of older adults, 57% were female. This is a notable increase from only 5 years prior where the older adults made up 1.5 million (11%) people living with HIV on treatment (Figure 1). The growth in the relative and absolute population of older adults living with HIV reflects both new clients initiating treatment in this age group and clients who have been part of the PEPFAR programme in prior years ageing into this cohort, with the latter accounting for a larger portion of the increase [2]. Among countries supported by PEPFAR, there is considerable variation in the proportion of the cohort that is older adults, with ranges from 7% to 30% across cohorts in 2021 [3]. While this proportion is lower than in many North American and western European cohorts, where older adults comprise nearly 50% of the treatment population, the absolute number of older adults living with HIV in PEPFAR‐supported countries is three times higher [4] than those in North America and western Europe.

Since 2015, the World Health Organization has recommended differentiated service delivery (DSD) models for HIV treatment to support “treat all” and in recognition of the diversity of needs of people living with HIV [5, 6, 7]. DSD for HIV has been defined as a person‐centred approach that adapts HIV services to meet the needs and expectations of people living with and at risk of acquiring HIV while acknowledging the constraints of the heathcare system. Adaptations for specific populations have addressed specific challenges, including stigma and discrimination, psychosocial support needs and provision of care outside of traditional clinical venues [8, 9, 10, 11]. Endorsed by PEPFAR and the Global Fund [12], DSD has been included in national HIV guidelines and implementation has been scaled up, particularly in high‐burden HIV countries. There has also been a concurrent focus on a life‐course approach in HIV programmes. This life‐course approach to DSD for HIV treatment has focused on earlier life phases—childhood and adolescence, families, and supporting sexual and reproductive health during childbearing years. Further, DSD for key populations—including men who have sex with men, people who inject drugs, sex workers and transgender people—has been designed to support improving HIV outcomes in these populations. Currently, there are no DSD models specifically designed for older adults, a rapidly growing cohort of individuals living with HIV.

In this commentary, we argue that with an ageing population of people living with HIV on treatment, health service providers and organizations acknowledge their unique challenges and consider developing DSD for HIV treatment models for older adults. We present common challenges faced by older adults living with HIV and suggest adaptations that might be considered and areas of research to ensure person‐centred care for the ageing cohort of people living with HIV.

## 2 DISCUSSION

### 2.1 HIV and ageing in the PEPFAR programme

Older adults have better treatment outcomes as measured by treatment continuity and viral suppression compared with younger people in the PEPFAR programme. Older adults living with HIV have the lowest proportion of loss to follow up with less than 2% experiencing an interruption in treatment when established on ART in a given quarter globally [13]. Older adults living with HIV also have the highest viral suppression rates with over 99% of clients [13] in the age group with viral load results available being suppressed (defined as VL<1000 c/ml). Thus, as effective therapies have improved the lifespan of people living with HIV, programmes can strive to enhance the quality of life as individuals age by tailoring services unique to this age group [14].

### 2.2 An ageing treatment cohort with specific health needs

Although older adults living with HIV may not need intensive, specific, support for HIV treatment compared with the specific counselling needs of younger people, they may have social and mental health needs that warrant consideration in DSD models. Three specific health challenges should be considered for older adults living with HIV:

1. High prevalence of co‐morbidities and associated treatments

Older adults bear a significant burden of non‐communicable diseases, and some studies document a greater prevalence of co‐morbidities in people living with HIV compared to those without HIV, including in African cohorts [15, 16, 17]. Many individuals with HIV have multiple co‐morbidities with some of the most common being diabetes, hypertension, obesity and renal insufficiency [18]. Additionally, older adults living with HIV are at increased risk of severe outcomes from both the long‐term complications of HIV treatment itself and other infectious diseases, including COVID‐19 and tuberculosis; the morbidity risk for these conditions may be additive. Being on multiple drugs related to co‐morbidities can create additional challenges for individuals living with HIV, including drug–drug interactions, adherence requirements for multiple medications and differing management requirements for chronic diseases [19]. Drug dispensation schedules for medicines other than antiretroviral therapy (ART) may differ significantly and the differences in frequency of required clinical and laboratory monitoring needed for other conditions can make management of these conditions more complicated, requiring multiple visits to healthcare facilities. There is a robust discussion in the international HIV treatment community about how best to build capacity within the healthcare system to provide holistic care for chronic diseases and provide integrated services for those with multiple co‐morbidities, including HIV. As countries determine their best response, policymakers and healthcare providers may be able to leverage various PEPFAR‐supported platforms—such as remote training modules for healthcare workers providing DSD, electronic medical records and drug dispensation tools—to achieve integrated service delivery to older adults.

2. High prevalence of geriatric syndromes

Older individuals have an age‐associated decline of physiological reserve and function and intrinsic capacity, which include sensory (vision and hearing), nutrition, mobility, depressive symptoms and cognitive decline, resulting in increasing vulnerability to a variety of stressors, including co‐morbidities. This has been called frailty in the medical literature and an increasing body of work identifies frailty as an independent risk factor for mortality and morbidity in both high resource and resource‐limited contexts [20, 21, 22]. Frailty is prevalent in the few studies that have evaluated it in Africa being more common in women, with a similar prevalence in people living with HIV and in those without but may appear earlier in individuals with HIV [23, 24, 25, 26].

Cognitive impairment is common among people living with HIV affecting up to 50% of individuals, including those who are on effective ART [27, 28, 29, 30]. In older adults, cognitive impairment may complicate treatment especially when there are multiple co‐morbidities [31]. Older adults commonly experience mental health challenges, both related and unrelated to their HIV status, including depression, isolation and loneliness [32]. Depression is a common neuropsychiatric comorbidity and is associated with “unhealthy ageing” characterized by accelerated neurocognitive decline [33]. The changes to daily life related to COVID‐19 restrictions are also likely to have further exacerbated many of these factors. Loss of spouse, income and other life‐changing events may further impact the mental health and wellbeing of these individuals.

Screening for frailty and other geriatric syndromes is recommended by many authorities but there is no consensus on the timing of screening and screening tools. Tools must be validated for the particular setting in which they are used [21, 22] and there are few studies evaluating evidence‐based interventions to address these geriatric syndromes to scale in order to improve outcomes in this vulnerable group. DSD for older adults could be designed to account for culture‐specific interventions and ageing‐related geriatric syndromes most relevant to their populations.

### 2.3 Where to from here

The provision of HIV treatment globally has been hailed as an unprecedented public health victory and the first chronic disease model scaled up in resource‐limited settings. Cornerstones of HIV treatment programmes have been simple therapies, task‐shifting and decentralization. Service delivery adaptations to the “building blocks” of services delivery—the frequency of services, the location of services and the package of services provided should be designed to address the specific challenges related to ageing. For example, where DSD for HIV treatment has reduced clinical consultations for many people by separating clinical consultations from drug refills, an older adult with co‐morbidities may require different types of evaluations and monitoring to address their health needs.

1. Apply the key enablers from DSD for HIV treatment to expand integration

As outlined by Bygrave et al., DSD for HIV has been enabled by “simplified algorithms, optimized formulations, secure drug supply and strengthened monitoring and evaluation systems” [34]. In addition, the removal of user fees has supported improved uptake and access to HIV treatment and linkage to HIV care already has promoted linkage to overall healthcare use and access [35]. Management of comorbid conditions and linkage to community‐based services for social support will likely be an important component of care provision. Even if all aspects of the health of an older individual cannot be addressed with the public health approach, certain conditions that are highly prevalent and amenable to an algorithmic approach to management and could be executed by providers with fewer years of formal medical training can result in substantial gains in further decreasing morbidity and mortality. Such conditions may include well‐controlled hypertension [36] and diabetes. Mechanisms used to procure drugs for HIV can be extended to diagnostics and treatments for these conditions as well.

2. Adapt or develop screening tools and treatment algorithms for common co‐morbidities validated in settings with high HIV burdens

Screening for cognitive disorders and mental health issues may be important in this population, and screening tools appropriate for the cultural and age context may need to be developed or adapted. These tools will need rigorous validation in the population in which they are deployed. The “trans diagnostic approach” in which it is recognized that mental health disorders often co‐occur and may have a shared underlying pathology has been helpful for the diagnosis and treatment of mental illness in resource‐limited settings and would need to be adapted to include identification and treatment of age‐related cognitive disorders [37, 38].

3. Follow country‐based innovations where HIV services are evolving and adapting to changing needs

There are emerging nascent examples from countries to provide more person‐centred care, primarily to improve the integration of common non‐communicable diseases. In South Africa, the Central Chronic Medicines Dispensing and Distribution (CCMDD) programme pre‐packs medications for chronic diseases, including HIV, hypertension and diabetes, for distribution through health facilities and community pick‐up points. More than two million clients are provided with medications pre‐packed by CCMDD, including 12% who are receiving ART plus other non‐communicable disease medications [39]. During COVID‐19, emphasis was placed on expanding the proportion of people collecting refills from community pick‐up points [10]. The investments made in healthcare worker training, capacity building, creating and modifying algorithms for care could all be applied to the wider health sector and bring a chronic disease care model to many countries that do not currently have one.

In Ethiopia, there is strong interest in the ministry of health to leverage the systems and the DSD capabilities developed in the PEPFAR platform to support the management of chronic co‐morbidities among people on ART. The country was early to adopt 6‐monthly ART refills in 2017 [40], subsequently offering additional DSD for HIV treatment models and accelerating access to extended ART refills to specific populations, including pregnant and breastfeeding women and newly initiated people of treatment in response to COVID‐19 [10]. To support the management of chronic diseases, it is anticipated that the experience gained from these successes in HIV treatment, including establishing national guidelines, technical working groups, scaled training and mentorship models, task shifting among health workers, data monitoring and use, and continuous quality improvement practices for HIV treatment, will provide a solid foundation for next steps with chronic disease management for older adults with HIV.

4. Increase research, including implementation science and qualitative research, that focuses on service delivery for older adults living with HIV

As countries and implementers innovate and adapt HIV services to the challenges of older adults, implementation research will be critical to understanding what works. Outcome measures, such as viral suppression and treatment continuity, are already established, but there may be other outcomes that are important to measure, including outcomes of co‐morbidities. Quality of life, “loss of frailty” and community connectedness may be useful to measure. As individuals live longer, it will be important to compare mortality and co‐morbidities to the non‐HIV populations. These efforts have the potential to improve the health and wellbeing of the general population.

Most older adults in treatment programmes are “successful” patients: they have been on effective therapy, often for decades, and have experienced changes in ART regimen and delivery systems. These individuals have successfully navigated HIV treatment and may be a valuable resource for programmes. These older individuals may be more treatment literate with respect to HIV than their younger counterparts, but their treatment literacy needs are poorly understood. Treatment literacy is associated with positive outcomes for HIV and several chronic conditions, including heart failure and diabetes [41, 42, 43], such as the need for age‐appropriate vaccines and falls prevention. The experience of older adults in PEPFAR programmes is poorly documented, and qualitative research in this area can inform how to provide the best information and care. More data are needed on the experience of older adults in treatment in PEPFAR settings and an effort should be made to harness the experiences of these individuals to improve care for all.

## 3 CONCLUSIONS

It is time for HIV programming to include specific DSD for HIV treatment models to address the needs of an ageing HIV cohort. With global decreases in new infections and continual increases in the number of people living with HIV on treatment, an ageing HIV cohort is an indicator of the huge progress and success of the HIV response. Building on experiences of adapting services, HIV services for specific populations, such as adolescents and key populations, acknowledging the realities of an ageing cohort and learning how to best provide person‐centred HIV care that will improve the quality of life of an older person living with HIV will be important.

The HIV needs of older adults are not limited to those living with HIV who are established on treatment. Older adults also require access to HIV prevention with their sexual health needs addressed beyond their reproductive years. Further, as the number of people living with HIV who do not know their status continually declines, HIV testing will be important for those previously not diagnosed and in this older cohort.

The initiative to care for the whole older adult will need commitment from global funders and in close partnership with ministries of health to achieve the best outcomes. As programmes are being implemented, care needs to be taken to develop outcome measures of success, document outcomes and successful models in the field so that they can be rapidly disseminated around the world to keep up with the pace of the ageing HIV population. Importantly, this would also align with the agreed‐upon UN sustainable development goal (SDG 3) of “ensuring health lives and promote well‐being for all at all ages” [44].

## COMPETING INTERESTS

AG is a deputy editor of the *Journal of the International AIDS Society*.

## AUTHORS’ CONTRIBUTIONS

AG and CG conceptualized the article. CG wrote the first draft. SV reviewed PEPFAR global and national data on ageing. Insight from Ethiopia was provided by MPS and from South Africa was provided by AG. All authors edited and approved the final manuscript.

## FUNDING

AG is supported by the Bill and Melinda Gates Foundation (INV002610).

## DISCLAIMER

CG, SV and MPS wrote this in their capacity as US government employees; the views expressed are their own and should not be construed to represent the positions of the Department of State, the Centers for Disease Control or the Department of Health and Human Services.
